# Single-shot X-ray and near-infrared (NIR) dual-mode fusion imaging based on bifunctional NIR scintillators

**DOI:** 10.1038/s41377-025-01898-8

**Published:** 2025-09-11

**Authors:** Peng Ran, Lurong Yang, Juan Hui, Yirong Su, Zeng Chen, Haiming Zhu, Cuifang Kuang, Xu Liu, Yang (Michael) Yang

**Affiliations:** 1 State Key Laboratory of Extreme Photonics and Instrumentation, College of Optical Science and Engineering, Zhejiang University, Hangzhou, 310027 China; 2 Jiaxing Key Laboratory of Photonic Sensing & Intelligent Imaging, Intelligent Optics & Photonics Research Center, Jiaxing Research Institute of Zhejiang University, 314000 Jiaxing, China; 3 Key Laboratory of Excited State Materials of Zhejiang Province, Department of Chemistry, Zhejiang University, 310027 Hangzhou, China

**Keywords:** Imaging and sensing, X-rays

## Abstract

X-ray and near-infrared (NIR) imaging are two well-established noninvasive imaging techniques, whose fusion often delineates a more complementary view of the subject. In this study, we introduce an innovative dual-mode imaging approach using a NIR scintillator, functioning both as a conventional scintillator for X-ray imaging and as a light source for NIR imaging. Our method facilitates the concurrent acquisition and registration of X-ray and NIR images in a single X-ray shot, eliminating the need for additional hardware beyond that of a standard X-ray imaging system. We have successfully synthesized an ytterbium-doped perovskite NIR scintillator using a water-based scalable process, which exhibits a pronounced scintillation emission at 980 nm, suggesting the presence of a potential quantum cutting effect. The experimental results underscore the enhanced capabilities in visualizing features typically elusive in standard X-ray images, such as the vascular network in a human palm. Besides, our method can effectively separate the X-ray and NIR signals, which is a common issue with recently developed multi-band detectors that suffer from superimposed electrical signals. This separation is achieved by designing a NIR-Visible dual-band scintillator that channels the X-ray and NIR characteristics into distinct emission pathways, thus avoiding any potential interference between the two imaging modalities. This study presents a novel strategy for harnessing the synergistic information from X-ray and NIR photons, enabled by the simple yet effective design of a NIR X-ray scintillator. This advancement might hold the potential to broaden the application scope of conventional X-ray imaging, enhancing its diagnostic and analytical capabilities.

## Introduction

**Fig. 1:**
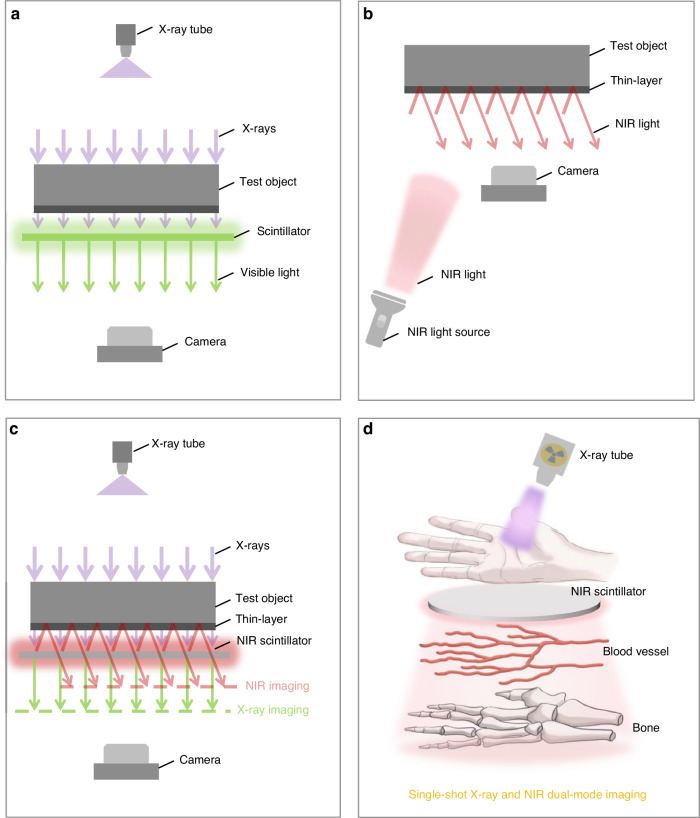
Schematic diagrams of X-ray and NIR dual mode imaging. **a** Schematic illustration of conventional X-ray imaging system. **b** Schematic illustration of conventional reflective NIR imaging system. **c** Schematic illustration of X-ray and NIR dual-mode imaging enabled by bifunctional NIR scintillators. **d** Schematic illustration of X-ray and NIR dual-mode fusion image of a human palm showing both bones and vessels

X-rays are essential in non-invasive medical and industrial diagnostics^1–3^, especially for visualizing materials with relatively high atomic numbers and densities that can efficiently attenuate X-rays^4–6^. In a standard configuration (Fig. 1a), X-rays traverse objects like human palms, creating contrast for dense tissues such as bones. Concurrently, Near-infrared (NIR) photons offer an alternative to non-invasive imaging thanks to their capacity to penetrate opaque materials like human tissues^7–11^. In a typical reflective NIR imaging configuration (Fig. 1b), NIR photons penetrate the skin, providing imaging contrast for blood vessels by exploiting the optical absorption of hemoglobin^12^. The distinct excitation mechanisms of X-ray and NIR photons render them complementary in the field of noninvasive imaging^13–15^. The amalgamation of X-ray and NIR imaging modalities can thus provide a more holistic diagnostic and inspection insight.

Detecting NIR photons diffused by tissues is an imaging technique with significant potential in the biomedical field^16–19^. However, this method often requires in vivo experiments with injected NIR emitters and long-term scanning imaging techniques. In contrast, our research emphasizes the non-invasive imaging properties of X-ray and NIR, integrating their standard applications to offer a more comprehensive imaging view for a variety of applications. A straightforward approach for dual-mode imaging involves the independent acquisition of X-ray and NIR images, followed by their fusion. However, this method encounters challenges in image registration and lacks the capability for real-time imaging^20^. The development of multimodal detectors based on materials responsive to multiple bands presents a potential solution^21,22^, but it has inherent limitations as well. Firstly, there are often conflicting demands for the optimization of devices and materials for these two detectors. For instance, X-ray detectors require very thick absorbers, in contrast to the thin NIR detectors because of its much larger absorption cross-section^23–26^. Secondly, the superimposed electrical signals generated by X-ray or NIR photons are impossible to decouple, making it unbale to image the objects with overlapped X-ray and NIR features^27,28^. Lastly, even with a unified detector, the dual-mode imaging still requires both X-ray and NIR light sources, potentially increasing the complexity and cost of the instruments, and introducing aberrations in the fusion image due to non-coincident illumination^29^.

To address those limitations, we introduce an innovative dual-mode imaging approach utilizing NIR scintillators. This scintillator fulfills a dual role, functioning both as a conventional scintillator for X-ray imaging and as an illumination source for NIR imaging (Fig. 1c). This approach enables the simultaneous acquisition of X-ray and NIR images in a single X-ray shot, without the need for additional hardware beyond that of a typical X-ray imaging system (Fig. 1d). Moreover, our scintillator-based strategy can effectively decouple X-ray and NIR signals, thereby overcoming the issue of signal superimposition encountered in previous multi-band detectors. By integrating a NIR-Visible dual-band scintillator, the resulting fused images clearly delineate distinct X-ray and NIR features at a single shot of X-ray.

## Results

Theoretical light yield in scintillators is inversely proportional to the band gap^30^, suggesting a higher potential for less-explored NIR scintillators than conventional counterparts. Besides, NIR scintillators may achieve the quantum cutting effect^31^, potentially doubling the limit. This study advocates for the use of metal-halide perovskite based NIR scintillators due to their exceptional photophysical properties, including a large X-ray absorption cross-section, high emission efficiency, and material adjustability^32–34^. Recently, ytterbium (Yb)-doped perovskite quantum dots have demonstrated significant potential in NIR LEDs^35^, and solar concentrators^36^. Although Yb-doping in CsPbCl_3_ single crystals has proven effective in achieving quantum cutting effects under ultraviolet excitation^37^, a large-scale synthesis method is still needed for fabricating large-area scintillation screens.

**Fig. 2:**
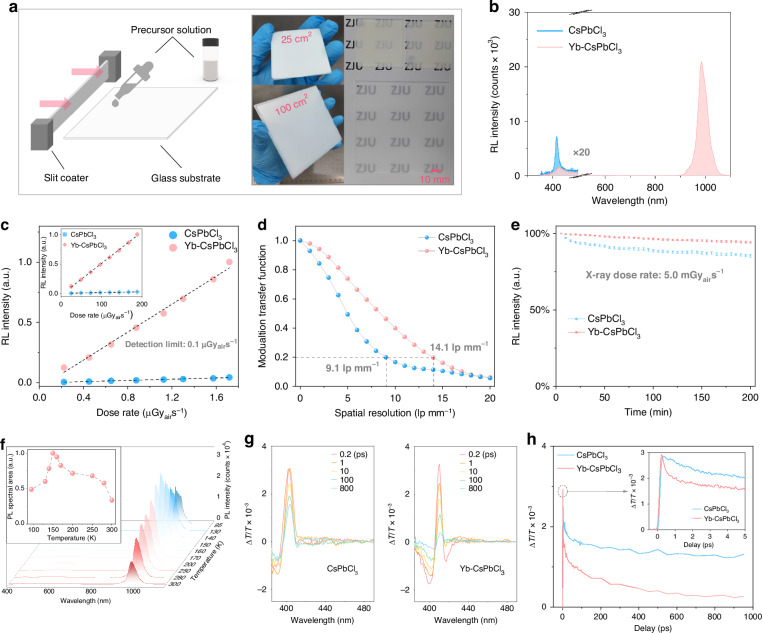
Preparation and characterization of Yb-CsPbCl_3_ NIR scintillators. **a** Schematic diagrams of the preparation process of Yb-CsPbCl_3_ scintillator screens. **b** RL spectra of CsPbCl_3_ and Yb-CsPbCl_3_ scintillators. **c** The RL output of CsPbCl_3_ and Yb-CsPbCl_3_ scintillators of same thickness at different X-ray excitation intensities. **d** MTF curves of the X-ray images obtained from CsPbCl_3_ and Yb-CsPbCl_3_ scintillators of same thickness. **e** RL intensity changes of CsPbCl_3_ and Yb-CsPbCl_3_ scintillators under 200 min continuous X-ray illumination. **f** PL spectra of Yb-CsPbCl_3_ powders at different temperatures. The inset shows the normalization integral area of the PL spectra. **g** TA spectra of CsPbCl_3_ and Yb-CsPbCl_3_ excited by a fs laser at 350 nm. **h** TA kinetics of the photobleaching (~405 nm) for CsPbCl_3_ and Yb-CsPbCl_3_, respectively

In this study, we have developed a facile and eco-friendly synthesis of Yb-doped CsPbCl_3_ microcrystalline powders on a large scale, employing water as the solvent. These microcrystals exhibit the maximized radioluminescence (RL) intensity with a Yb/Pb feeding ratio of 75 mol% (Figure S1). Subsequently, we incorporated these microcrystals into a solution of polymethyl methacrylate, which can be blade-coated onto glass or plastic substrates to create the dual-mode NIR scintillator screen (Fig. 2a).

Figure 2b illustrates the RL spectra of undoped and Yb-doped CsPbCl_3_ scintillators. Undoped CsPbCl_3_ exhibits a luminescence peak at 416 nm, indicating band-edge excitonic emission. In contrast, Yb-doped CsPbCl_3_ displays minimal band-edge luminescence but intense broadband NIR luminescence at 984 nm, attributed to efficient excitation energy transfer from CsPbCl_3_ to Yb, causing strong quenching of band-edge luminescence^38^. Yb-CsPbCl_3_ scintillators have a substantial Stokes shift, avoiding the adverse effects of self-absorption in conventional perovskite scintillators^39^. Figure 2c shows the light output of undoped and Yb-doped CsPbCl_3_ scintillators at different X-ray dose rates, revealing a favorable linear response. The RL intensity of Yb-CsPbCl_3_ is tens of times higher than undoped CsPbCl_3_. The lowest detection limit for Yb-CsPbCl_3_ is 0.1 μGy_air_s^−1^. The Yb-CsPbCl_3_ scintillator screen delivers a resolution of approximately 14.1 lp mm^−1^ in our X-ray imaging system, surpassing that of the CsPbCl_3_ (9.1 lp mm^−1^) of the same thickness (Fig. 2d and S2). The scintillator screen captures clear images of common objects, establishing the framework for subsequent dual-mode imaging experiments (Figure S3).

Figure 2e illustrates a mere 5.8% decline in RL output following a continuous 200-min exposure to X-rays (dose rate: 5.0 mGy_air_s^−1^) for Yb-CsPbCl_3_. In contrast, CsPbCl_3_ experienced a more substantial 14.6% degradation. This difference is likely ascribed to the enhanced defect tolerance of the NIR emission of Yb when compared to the band-edge excitonic emission of CsPbCl_3_. Under continuous high-energy X-ray irradiation, the soft lattice structure of perovskites readily gives rise to numerous defect states. These defect states serve as non-radiative recombination centers, leading to the degradation of luminescence performance. In Yb-CsPbCl_3_, however, the excited states are rapidly transferred to Yb sites (as shown in Fig. 2g, h) and photons are emitted through Yb energy levels. Owing to its parity-forbidden 4f-4f transition mechanism, Yb has intrinsic stability that enables it to resist external perturbations. As a result, Yb-CsPbCl_3_ exhibits better X-ray radiation stability compared to pristine CsPbCl_3_.

We conducted a comparison between our Yb-CsPbCl_3_ scintillator and the commercial scintillator LuAG: Ce. Figure S4 illustrates their absorption coefficients at various X-ray energies and attenuation efficiency at different thicknesses, revealing that Yb-CsPbCl_3_ is slightly better than LuAG: Ce in this aspect. Thickness-dependent relative light outputs were measured using the setup in Figure S5, revealing both scintillators reach maximum light output at 100 μm. Notably, Yb-CsPbCl_3_ consistently outperforms LuAG: Ce in terms of light output across the measurement range, as shown in Fig. S6. Using the standard commercial scintillator LuAG: Ce as a reference sample, we estimate the relative light yield of Yb-CsPbCl_3_ to be approximately 31, 223 photons MeV^−1^. This estimation is based on a comparison of the RL spectral areas of samples with equal thickness, under identical X-ray irradiation conditions. Additionally, the measurement system was cross-validated with other typical commercial scintillators, including CsI: TI, YAG: Ce, and BGO, with the obtained light yields matching the values reported in their datasheets. This confirms the validity of our measurement method. Figure S11 shows their RL spectra measured under the same conditions, along with the relative light yield calculated from the spectral area comparison. Figure S12 presents the time-resolved photoluminescence (TRPL) spectra of Yb-CsPbCl_3_ at room temperature (λ_em_ = 980 nm), excited by a 350 nm laser. Exponential fitting of the TRPL spectrum yields a decay lifetime of 2.13 ms.

We further explore the photophysical properties of Yb-CsPbCl_3_ through temperature-dependent photoluminescence (PL) and transient absorption (TA) spectroscopies. Figure 2f displays the steady-state PL spectra of Yb-CsPbCl_3_ powders across temperatures ranging from 95 to 300 K. The PL intensity exhibits an upward trend as the temperature decreases from 300 to 150 K, indicating reduced non-radiative recombination. Notably, at 150 K, the integrated PL intensity of the NIR region area is 3.5 times larger than that at 300 K. The measured PL quantum yield (PLQY) at 300 K is 44.5% (Figure S7), hinting at a potential increase to over 100% at 150 K (estimated to be 3.5 × 44.5%), assuming the absorption cross-section remains relatively insensitive to temperature^40^. This suggests the quantum-cutting effect as the underlying origin of the high NIR emission in Yb-CsPbCl_3_, which can be further supported by our ultrafast TA measurements. The femtosecond time-resolved TA exciton bleach-recovery kinetics captured on CsPbCl_3_ and Yb-CsPbCl_3_ films are displayed in Fig. 2g and S8. Both samples exhibit typical ground-state bleach and excited-state absorption. Significantly, Yb-CsPbCl_3_ demonstrates a notably quicker recovery than that of CsPbCl_3_. This observation suggests the presence of an additional carrier trapping channel, facilitating the energy transfer to Yb dopants. The TA kinetics were specifically monitored at the exciton bleach (XB) at around 405 nm, as illustrated in Fig. 2h. This analysis confirms that Yb-CsPbCl_3_ shows an ultrafast picosecond energy transfer process, clearly surpassing the decay kinetic of band edge exciton in the nanosecond scale^37,41^. The observation of this ultrafast process certainly ensures efficient energy transfer from the CsPbCl_3_ host to Yb dopants, resulting in subsequent NIR quantum-cutting emission. To further support that this ultrafast process involves energy transfer rather than Auger electrons, TA measurements of the Yb-CsPbCl_3_ samples were meticulously conducted under further reduced excitation intensities (Figure S9). They reveal that this picosecond ultrafast processes is independent on excitation intensity, ruling out the effects of Auger electrons^42^. These findings not only suggest potential strategies for enhancing NIR scintillation through the quantum-cutting effect but also lay the foundation for effective X-ray and NIR dual-mode imaging.

**Fig. 3:**
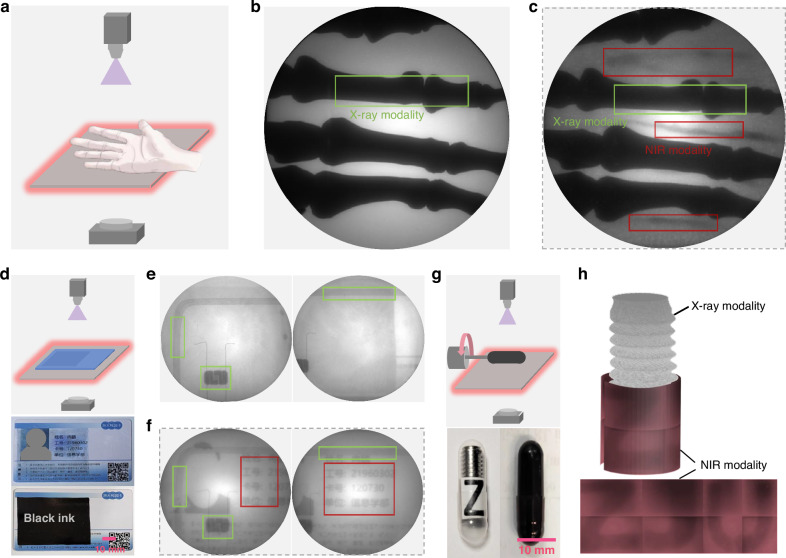
2D and 3D dual-mode imaging using NIR scintillator for enhanced visualization compared to conventional X-ray imaging using visible light scintillator. **a** Schematic representation of the dual-mode imaging applied to a human palm model. **b** The conventional X-ray imaging of a human palm showing the bare bones. **c** The dual-mode imaging of a palm showing both bones (X-ray modality) and vessels (NIR modality). **d** Schematic representation of the dual-mode imaging applied to a campus card. **e** The conventional X-ray imaging of a campus card showing the only coil and chip embedded within the card. **f** The dual-mode imaging of a campus card uncovering hidden information smeared in black ink. **g** Schematic representation of the dual-mode 3D imaging applied to metallic spring in a black capsule. **h** The reconstructed CT image of dual-mode 3D volumetric imaging of metallic spring in a black capsule with “ZJU” label

In this section, we validated our proposed concept of dual-mode imaging using the Yb-CsPbCl_3_ NIR scintillator screen and compared it with conventional X-ray imaging using visible light scintillators. Figure 3a demonstrates a comparative experiment on a human palm model, where traditional X-ray imaging only captures bone images (Fig. 3b) due to weak X-ray attenuation by blood vessels. In contrast, dual-mode imaging (Fig. 3c) provides contrast for both bones and blood vessels. Our approach, unlike other multimodal imaging strategies^43–45^, requires no additional hardware components and can instantly obtain both images simultaneously, which could be helpful for enhancing medical diagnosis efficiency. This method could also provide more information in some industrial inspection applications. As illustrated in Figure S10, we have compared the imaging of a spring-loaded capsule within a black box using both our NIR-X-ray dual-mode imaging and conventional X-ray imaging techniques. While both methods successfully imaged the capsule, only the dual-mode imaging was able to reveal details painted on the inner side of the black box. Figure 3d compares the imaging of a university ID card smeared with black ink. As illustrated in Figs. 3e, f, while both X-ray and Dual-mode imaging techniques could visualize the coil and chip embedded within the card, only the dual-mode imaging was capable of uncovering the text obscured by the black ink. The synergistic integration of both modalities significantly enhances the ability to detect and differentiate substances, making it a valuable tool for a wide range of applications.

The aforementioned demonstrations affirm the enhanced capabilities of our dual-mode imaging system in two-dimensional (2D) applications. We now extend the discourse to explore its implications in three-dimensional (3D) Computed Tomography (CT) imaging. The X-ray-NIR dual-mode 3D CT approach is expected to yield richer volumetric information compared to standard X-ray CT. However, the 3D reconstruction of dual-mode images requires precise temporal alignment, posing significant challenges for conventional methods that involve sequential acquisitions of corresponding X-ray and NIR images^46,47^. Our proposed concept addresses this challenge by capturing both X-ray and NIR images concurrently at each viewing angle. As depicted in Fig. 3h, this enables the 3D dual-mode CT imaging, where the X-ray modality delineates the metal spring within a black capsule, and the NIR modality reveals the “ZJU” label on a paper inside the capsule, demonstrating the ability to provide detailed volumetric information from both imaging modalities.

**Fig. 4:**
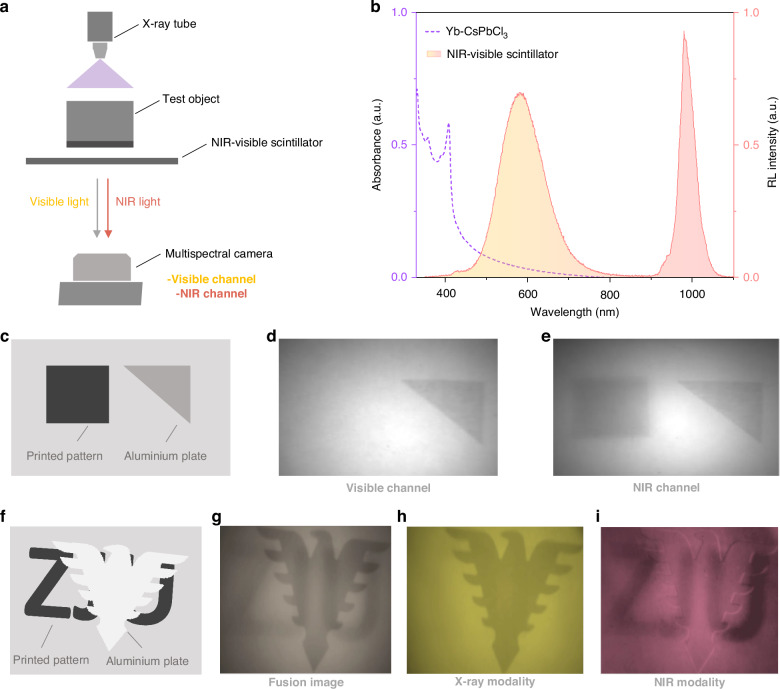
X-ray and NIR signal decoupling based on NIR-Visible dual-emission scintillators. **a** Schematic illustration of the X-ray and NIR signal decoupling based on NIR-Visible dual-emission scintillators. **b** RL spectrum of NIR-Visible dual-emission scintillator composed of Yb-CsPbCl_3_ and CsCu_2_I_3_. **c** Configuration of the test subjects-an aluminum plate and a black cardboard, positioned within a black opaque box, simulating complex imaging scenarios and assess the decoupling capability of the dual-mode system. **d** Visible channel image emulates the conventional X-ray imaging modality. **e** NIR channel image contains both X-ray and NIR modalities. **f** Schematic of the test subject, featuring a black cardboard with the inscription “ZJU” covered by an aluminum eagle. **g** The dual-mode fusion image showing an overlapping pattern of both aluminum eagle (X-ray modality) and “ZJU” inscription (NIR modality). **h** The sole X-ray modality image produced from the visible channel. **i** The sole NIR modality image was obtained by subtracting the X-ray modality image (Fig. 4i) from the fusion image (Fig. 4g)

The presented 2D and 3D dual-mode imaging examples highlight its superiority over conventional single-modality X-ray or NIR imaging, by providing richer information. In some intricate situations where X-ray and NIR signals intersect, it is essential for the multimode detectors to decouple the overlapping signals. In fact, this is particularly challenging with those recently developed dual-mode detectors that produce identical electrical outputs for both X-ray and NIR inputs^21^. To address this, we introduce and validate a method for signal decoupling in dual-mode imaging, using our NIR-Visible dual-band scintillator. As shown in Fig. 4a, the X-ray imaging modality is reflected exclusively through the visible scintillation channel, whereas both X-ray and NIR imaging modalities are captured by the NIR channel. By applying a straightforward image subtraction process, we can extract isolated X-ray and NIR imaging modality respectively.

The dual-band scintillator was prepared by mixing a NIR scintillator Yb-CsPbCl_3_ and a visible scintillator CsCu_2_I_3_. Figure 4b shows the RL spectrum of the dual-emission scintillator, where the absorption spectra of Yb-CsPbCl_3_ do not overlap with the emission of CsCu_2_I_3_. Figure 4c depicts the test subjects (an aluminum plate and a black cardboard), placed inside a black opaque box. Images from the visible and NIR channels of a multispectral camera are presented in Fig. 4d, e, respectively. Figure 4d solely displays the X-ray image of the aluminum originating from the CsCu_2_I_3_ scintillation, while Fig. 4e captures the X-ray and NIR dual-mode images of both aluminum plate and black cardboard originating from NIR scintillation of Yb-CsPbCl_3_. Subtracting Fig. 4d from Fig. 4e yields distinct X-ray and NIR images, enabling signal decoupling, which is crucial for scenarios where those two imaging modalities overlap. For example, in Fig. 4f, a black cardboard labeled with “ZJU” is stacked with an aluminum eagle, simulating such an intricate scenario. The fusion image produced from the NIR channel appears as an overlapping pattern of both the eagle and “ZJU” (Fig. 4g), whereas the X-ray image produced from the visible channel reveals only the aluminum eagle (Fig. 4h). By subtracting the sole X-ray image from the superimposed image, we can isolate the NIR image of the “ZJU” label (Fig. 4i). In summary, our NIR scintillator, when paired with a conventional visible scintillator, can exploit dual-band emissions to effectively decouple X-ray and NIR imaging modalities.

## Discussion

In summary, we present an innovative X-ray and NIR fusion imaging technique enabled by a dual-function NIR scintillator. This technique allows for the concurrent capture of images from both modalities, bypassing the need for complex image registration processes and an extra NIR illumination source. Utilizing an advanced ytterbium-doped perovskite NIR scintillator, we have demonstrated significantly enhanced imaging capabilities in both 2D and 3D dual-mode imaging configurations, compared to the traditional X-ray and NIR imaging. This approach synergistically harnesses the complementary strengths of X-ray and NIR photons, offering a promising avenue to broaden the applications of traditional X-ray imaging and to augment its diagnostic and analytical potential. Future progress may focus on improving light yield and resolution through scintillator light management strategies^48–50^ to enhance image quality. Applications could extend to biomedical diagnostics, industrial material analysis, and the preservation of cultural artifacts. Integration with AI-enhanced workflows or advanced optical systems could enable real-time dynamic multimode imaging and fusion image decoupling. By prioritizing scalable material development and fostering cross-disciplinary collaboration, this technology has the potential to redefine multimodal imaging across both scientific and industrial fields.

## Materials and methods

### Chemicals

Lead chloride (PbCl_2_, 99.9%), Cesium chloride (CsCl, 99.9%), and Ytterbium acetate tetrahydrate (YbC_3_H_6_O_2_^.^4H_2_O, 99.8%) were purchased from Aladdin. Cesium iodide (CsI, 99.9%), copper iodide (CuI, 99.95%), and Methanol (99.8%) were purchased from Aladdin. N, N-Dimethylformamide (DMF, 99.9%), and dimethyl sulfoxide (DMSO, 99.9%) were purchased from J&K Scientific. Polymethyl methacrylate (99%) is sourced from Kuer Chemical. Methylbenzene (AR) was purchased from Sinopharm Chemical Reagent Co. Ltd. Deionized water uses 5 L products of Wahaha Company. All reagents and solvents were used without further purification.

### Synthesis of CsPbCl_3_ and Yb-CsPbCl_3_ microcrystalline powder

The synthesis of CsPbCl_3_ microcrystalline powder followed the evaporative solvent crystallization method. Initially, 18.30 mg of CsCl and 30.23 mg of PbCl_2_ were dissolved in 400 ml of deionized water and stirred for 10 h at 30 °C until completely dissolved. The resulting solution was then transferred to a clean petri dish and allowed to evaporate slowly at 60 °C until the solvent completely evaporated. Subsequently, the dried material was annealed at 200 °C for 2 h, resulting in the formation of CsPbCl_3_ coarse powder. For Yb-doped CsPbCl_3_ powder, only ytterbium acetate tetrahydrate with the required Yb/Pb feed ratio is added to deionized water (e.g., 34.41 mg of YbC_3_H_6_O_2_·4H_2_O at 75 mol%). The subsequent steps remain identical to those for undoped CsPbCl_3_ powder.

### Synthesis of CsCu_2_I_3_ microcrystalline powder

The synthesis of CsCu_2_I_3_ powder utilized the anti-solvent method. Initially, a precursor solution was created by dissolving 2.5 mmol of CsI and 5 mmol of CuI in a 5 ml mixture of DMF and DMSO (in a 4:1 ratio), which was then stirred for 4 h at 60 °C. In a centrifuge tube, 2 mL of methanol was added as the anti-solvent, followed by the rapid injection of 500 μL of a precursor solution. This resulted in the immediate formation of a significant amount of white precipitate. Subsequently, the centrifuge tube was placed in a centrifuge and spun at a speed of 5000 rpm min^−1^ for 5 min. The supernatant was then discarded, and the residue was rinsed with methanol. This rinsing process was repeated more than three times, and the resulting precipitate was dried at 60 °C in a vacuum oven to obtain a coarse powder.

### Fabrication of scintillator screen

Initially, the Yb-CsPbCl_3_ coarse powder was ground into a fine powder using an agate mortar. A solution containing polymethyl methacrylate (PMMA) in toluene, with a concentration of 200 mg mL^−1^, was then prepared and stirred at 70 °C for 2 h. Subsequently, the Yb-CsPbCl_3_ fine powder was added to the PMMA-toluene mixture. The additional toluene solution was added to dilute the mixture. As Yb-CsPbCl_3_ is insoluble in toluene, thorough stirring was necessary to ensure a uniform dispersion and the formation of an emulsion. The prepared mixture was then evenly poured onto a thin glass substrate after adjusting the spatula of the blade coater to an appropriate height. The coating was performed at a speed of 5 mm s^−1^. Finally, the scintillation screen was obtained by placing the glass base in a dry environment until the toluene was completely volatilized, leaving behind the desired coating.

### Measurement of radioluminescence spectrum and intensity

A fiber-coupled fluorescence spectrometer (Ocean Optics QE PRO), an X-ray tube (Mini-X, Amptek Inc), and a quartz mold containing the scintillator powder sample were constructed into an experimental system. The RL spectral area can be calculated by integrating the intensity of the RL spectrum collected by a spectrometer.

### 2D X-ray imaging and dual-mode imaging system

The X-ray source utilized in this study was the Mini-X2 X-ray tube manufactured by Amptek Inc., with a tungsten target material and a maximum power output (P_max_) of 10 W. In this setup, X-rays initially passed through the imaging object and were then absorbed by the scintillator screen. Subsequently, the optical path underwent deflection via a reflector to mitigate the adverse effects of direct X-ray radiation on the camera. Finally, X-ray images were captured using a CMOS camera (Photometrics, Prime 95B). It’s worth noting that this experimental system also supports X-ray and near-infrared (NIR) dual-mode imaging, wherein the camera can simultaneously receive both X-ray and NIR imaging information.

### 3D dual-mode imaging system

Utilizing the same 2D imaging device described earlier, the measured object was positioned on an electric rotating table. X-ray irradiation occurred at intervals of every 10° rotation to acquire dual-mode projection images of the test object from various scanning angles. The filtered back projection method was employed to reconstruct a 3D image of the screw, while NIR information obtained from each projection was utilized for letter restoration. By integrating the X-ray and NIR images, a dual-mode 3D image was generated, providing comprehensive visual information about the test object.

## Supplementary information

Supplementary Figures S1-S18

## Data Availability

The data that support the findings of this study are available from the corresponding author upon reasonable request.
