# Supplementary material for: Single-shot X-ray and near-infrared (NIR) dual-mode fusion imaging based on bifunctional NIR scintillators
Source: Light Sci Appl. 2025 Sep 11;14:315. doi: 10.1038/s41377-025-01898-8 (PMC12426231; doi:10.1038/s41377-025-01898-8)
Supplement: Supplementary file 1 — Supplementary Figures S1-S18 [file 41377_2025_1898_MOESM1_ESM.docx]

**Supplementary Information for**

**Single-Shot X-Ray and Near-Infrared (NIR) Dual-Mode Fusion Imaging Based on** **Bifunctional NIR Scintillators**

Peng Ran^1,2^, Lurong Yang^1^, Juan Hui^1,2^, Yirong Su^1^, Zeng Chen^3^, Haiming Zhu^3^, Cuifang Kuang^1^, Xu Liu^1*^, Yang (Michael) Yang^1,2^*

^1^State Key Laboratory of Extreme Photonics and Instrumentation, College of Optical Science and Engineering, Zhejiang University, Hangzhou, 310027, China

^2^Jiaxing Key Laboratory of Photonic Sensing & Intelligent Imaging, Intelligent Optics & Photonics Research Center, Jiaxing Research Institute of Zhejiang University, 314000, Jiaxing, China

^3^Key Laboratory of Excited State Materials of Zhejiang Province, Department of Chemistry, Zhejiang University, Hangzhou, 310027, China

*Correspondence and requests for materials should be addressed to Yang (Michael) Yang [(yangyang15@zju.edu.cn)](mailto:(yangyang15@zju.edu.cn)) ; Xu Liu (liuxu@zju.edu.cn)


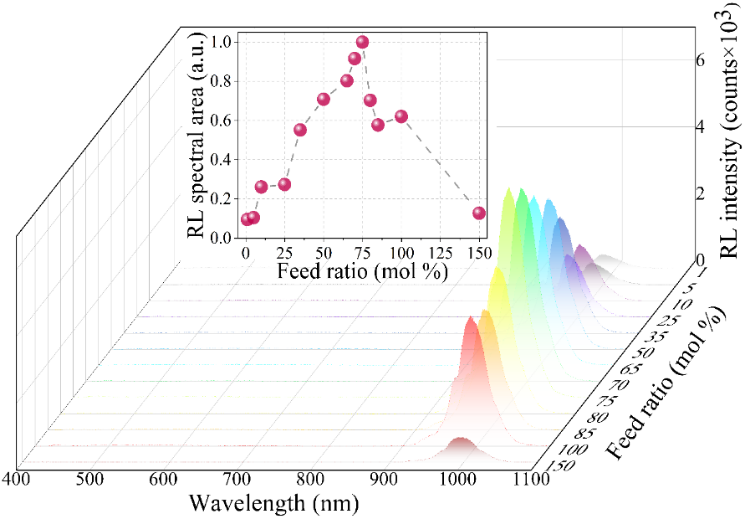


**Figure S1** RL spectra of Yb-CsPbCl_3_ synthesized at different Yb/Pb feed ratios. The illustration shows the integral area of the RL spectrum.

**
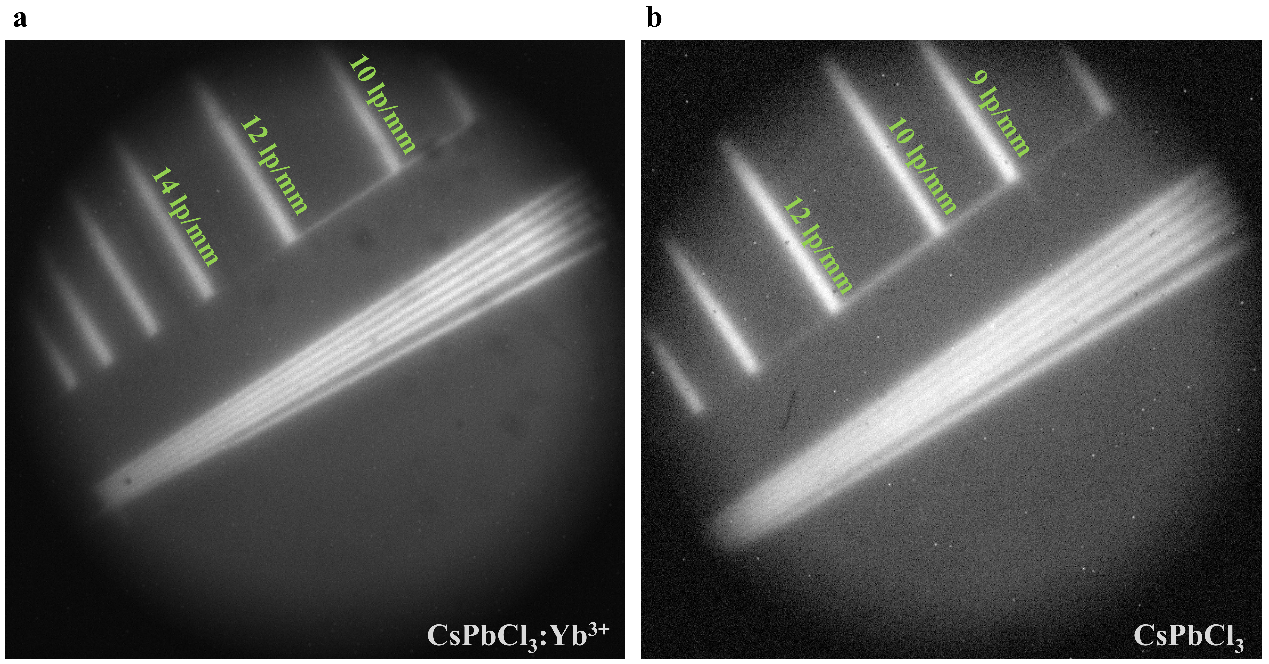
**

**Figure S2** The X-ray images of a standard spatial resolution test board obtained by the same thickness **(a)** Yb-CsPbCl_3_ **(b)** CsPbCl_3_ scintillators.


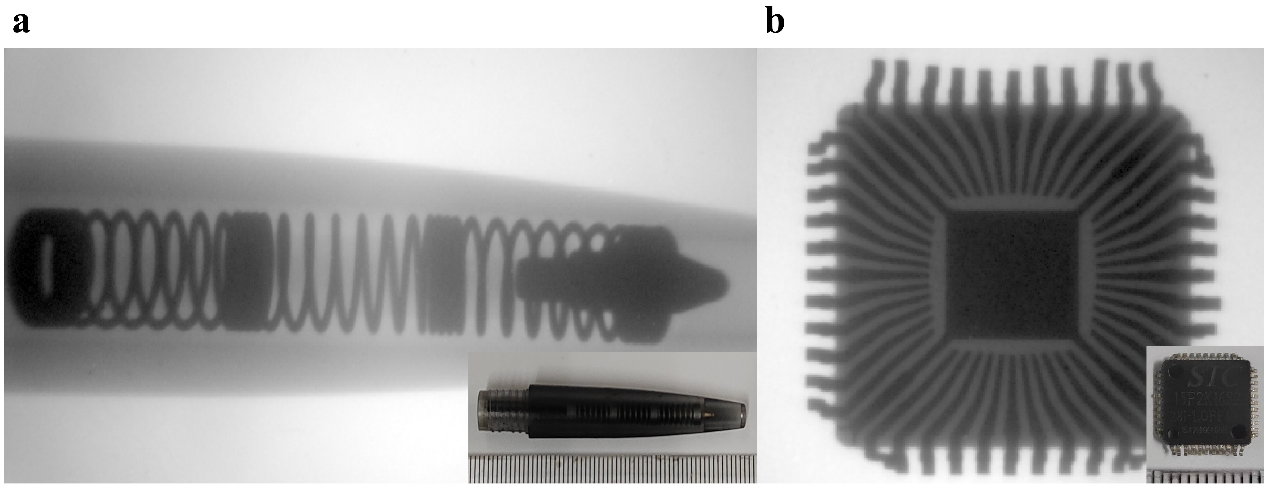


**Figure S3** The X-ray images of **(a)** a pen and **(b)** a chip using Yb-CsPbCl_3_ scintillator. The insets are photographs of them under visible light.


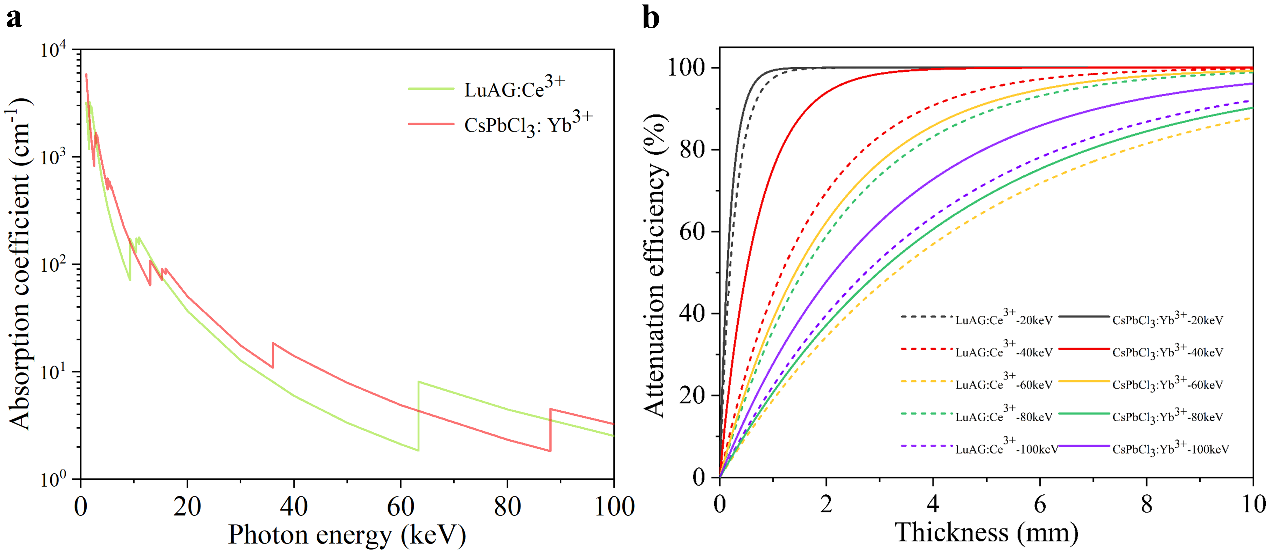


**Figure S4** **(a)** The absorption coefficients at various X-ray energies and **(b)** attenuation efficiency at different thicknesses of Yb-CsPbCl_3_ and LuAG: Ce.


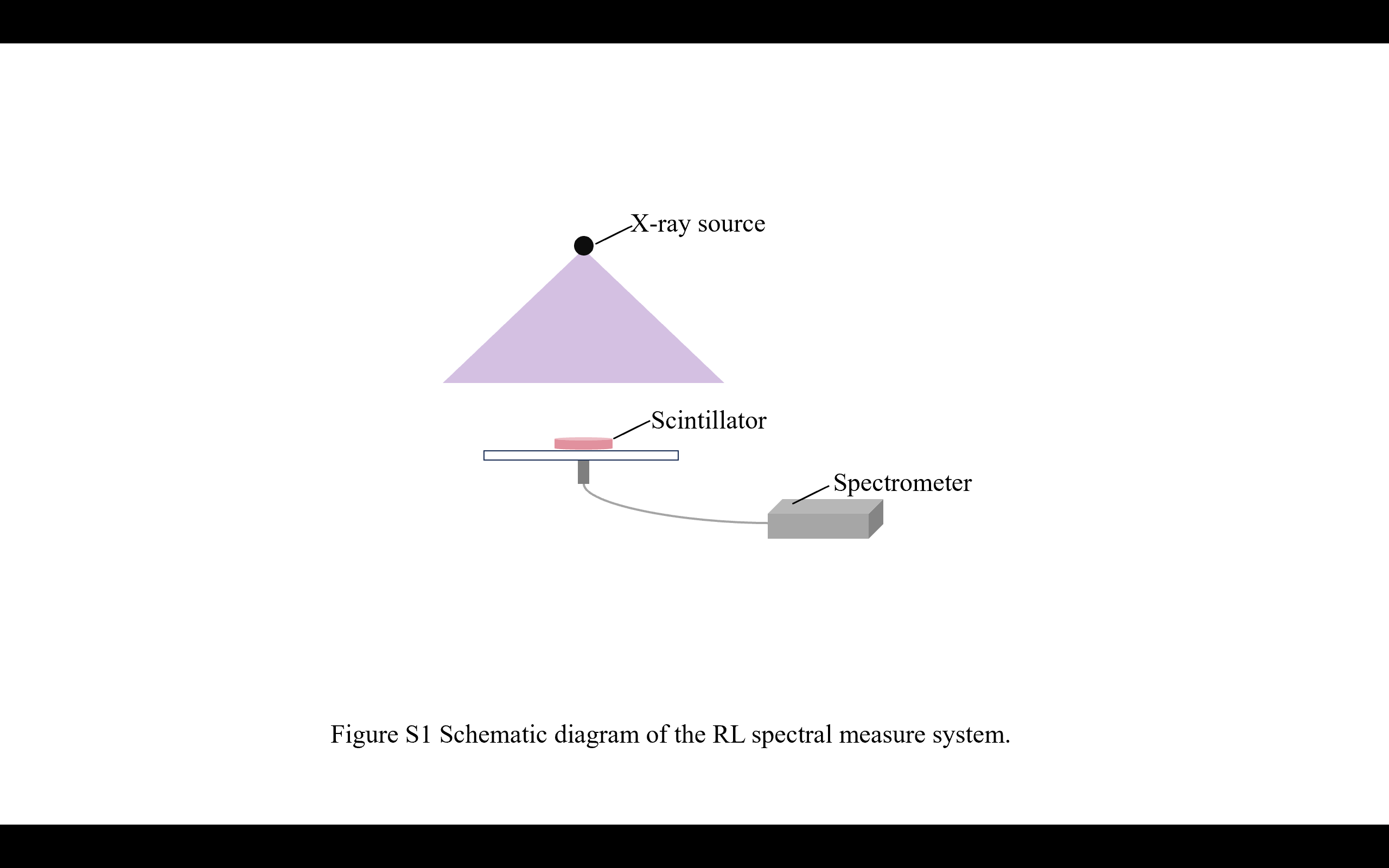


**Figure S5** Schematic diagram of the RL spectral measurement system.


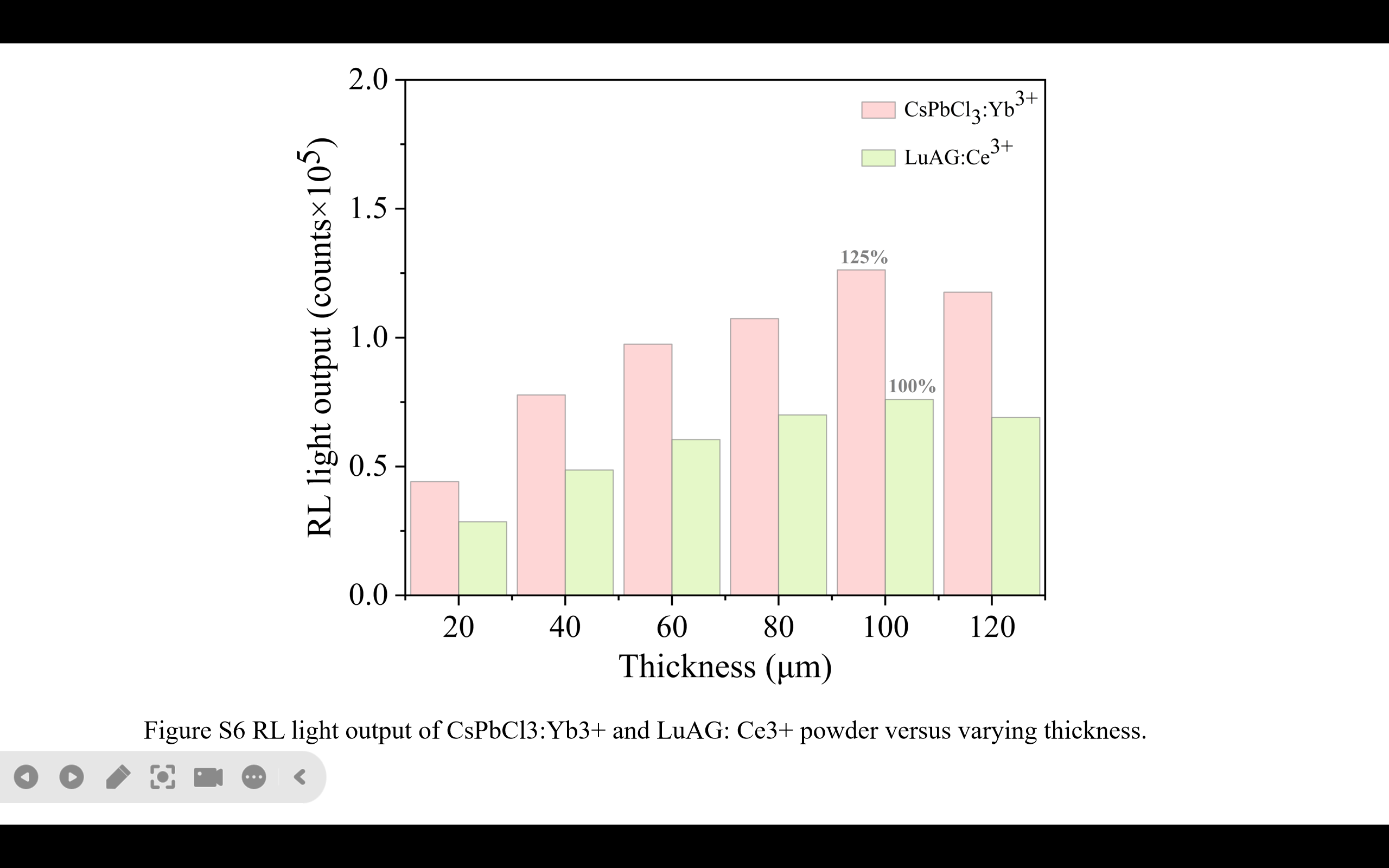


**Figure S6** RL light output of Yb-CsPbCl_3_ and LuAG: Ce powder versus varying thicknesses.


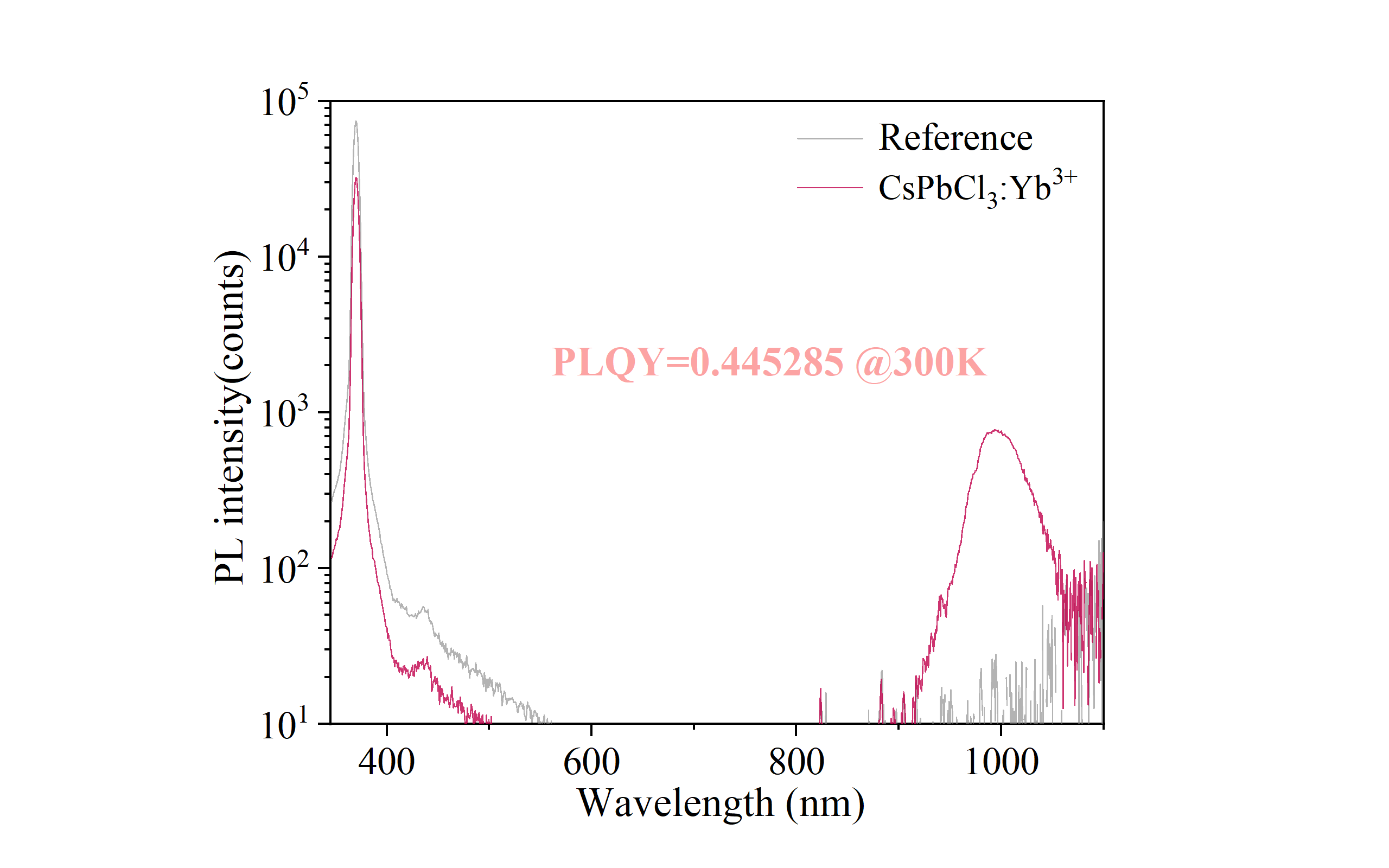


**Figure S7** The PLQY measurement of Yb-CsPbCl_3_ powder at 300 K.


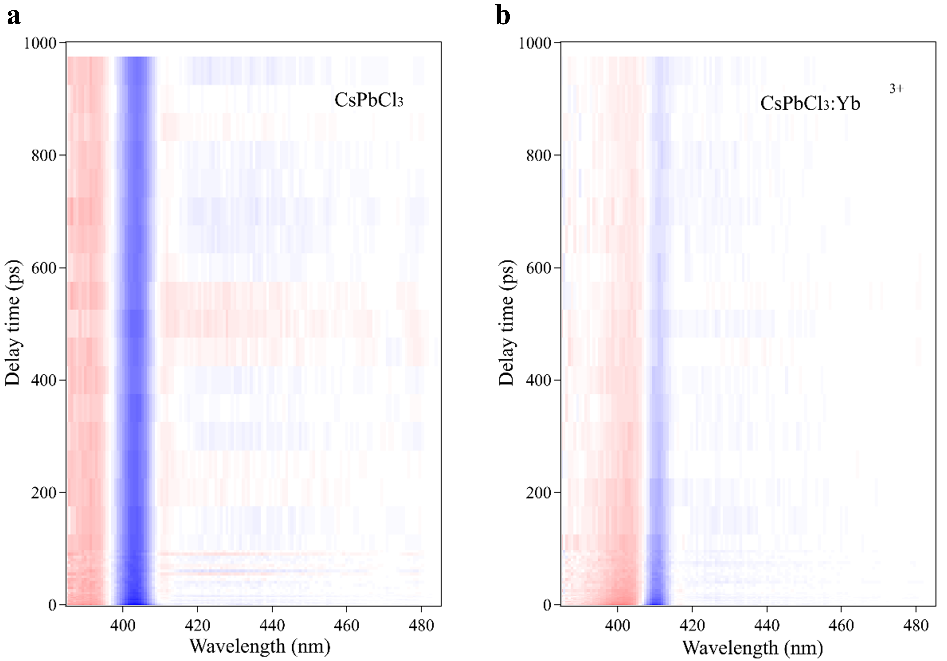


**Figure S8** Two-dimensional TA spectra of **(a)** CsPbCl_3_ and **(b)** Yb-CsPbCl_3_ films.


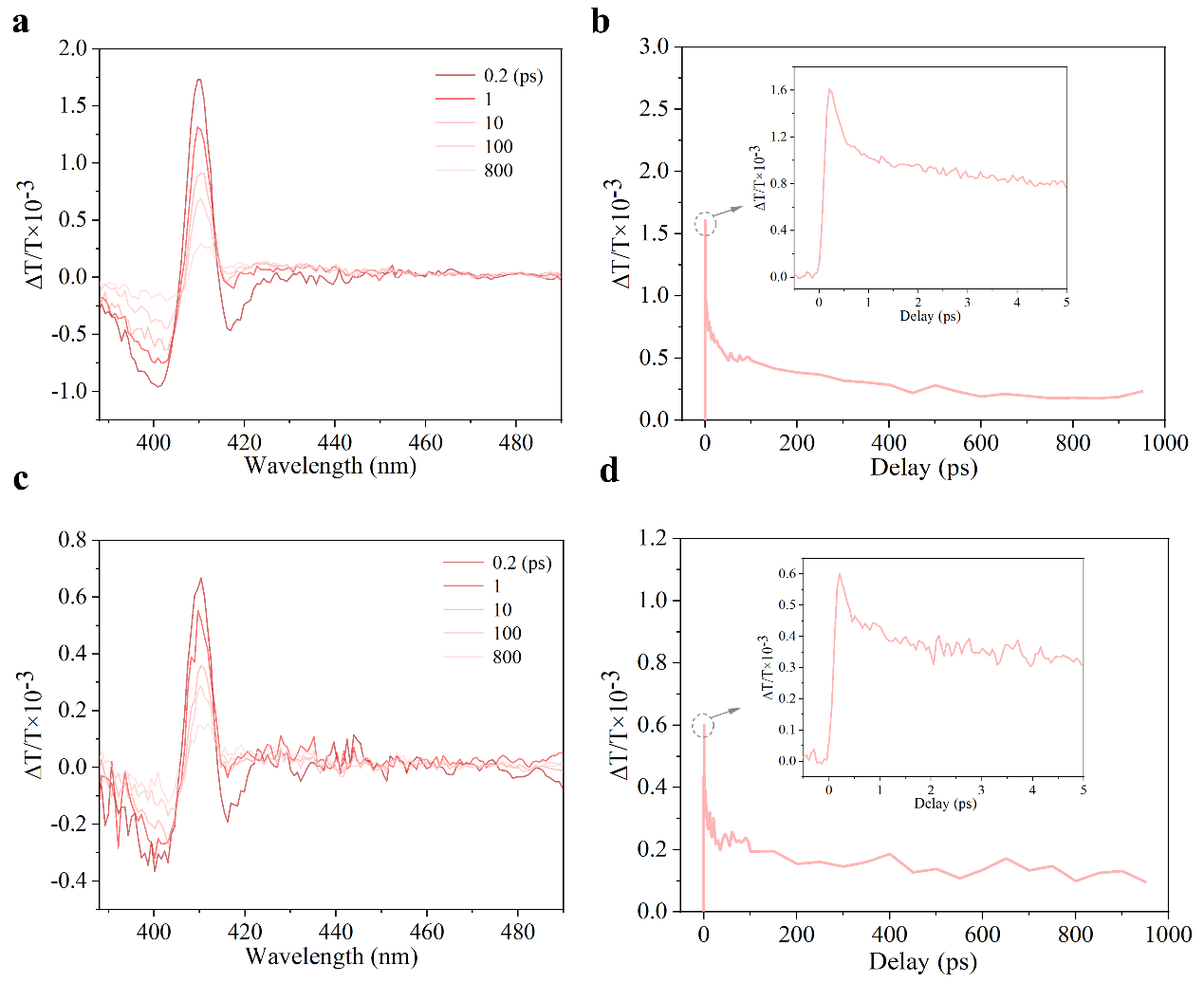
**Figure S9** TA spectra and kinetics monitored at 405 nm of Yb-CsPbCl_3_ following the excitation by a 350 nm pump pulse with energy density of **(a)(b)** around 4.0 μJ/cm^2^ **(c)(d)** around 2.0 μJ/cm^2^.


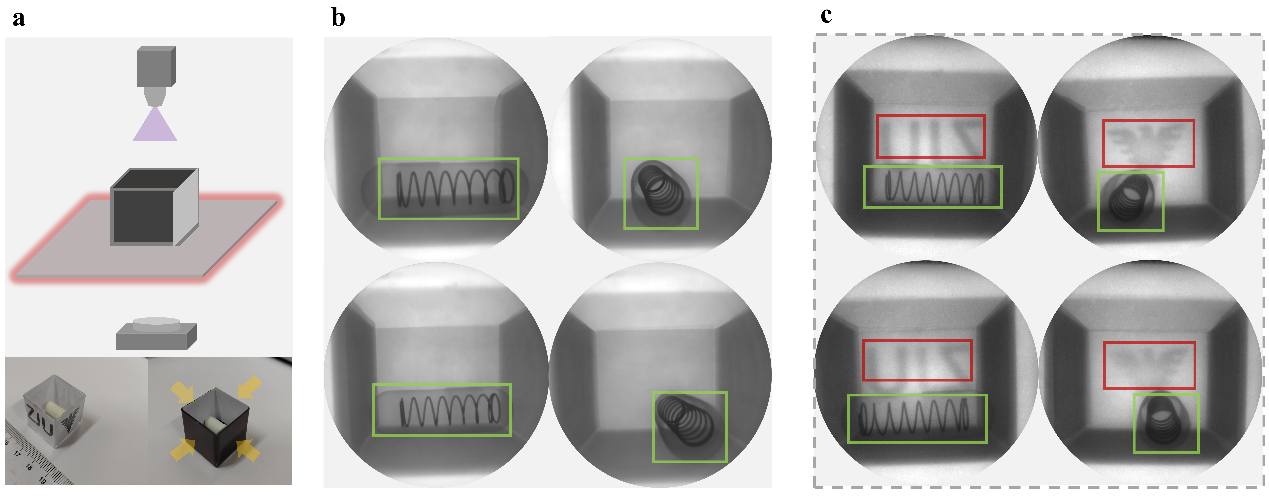


**Figure S10** **(a)** Diagram of dual-mode imaging of spring-loaded capsule within a black box. **(b)** The conventional X-ray imaging showing only the capsule (X-ray modality). **(c)** The dual-mode imaging showing both the capsule (X-ray modality) and details painted on the inner side of the black box (NIR modality).


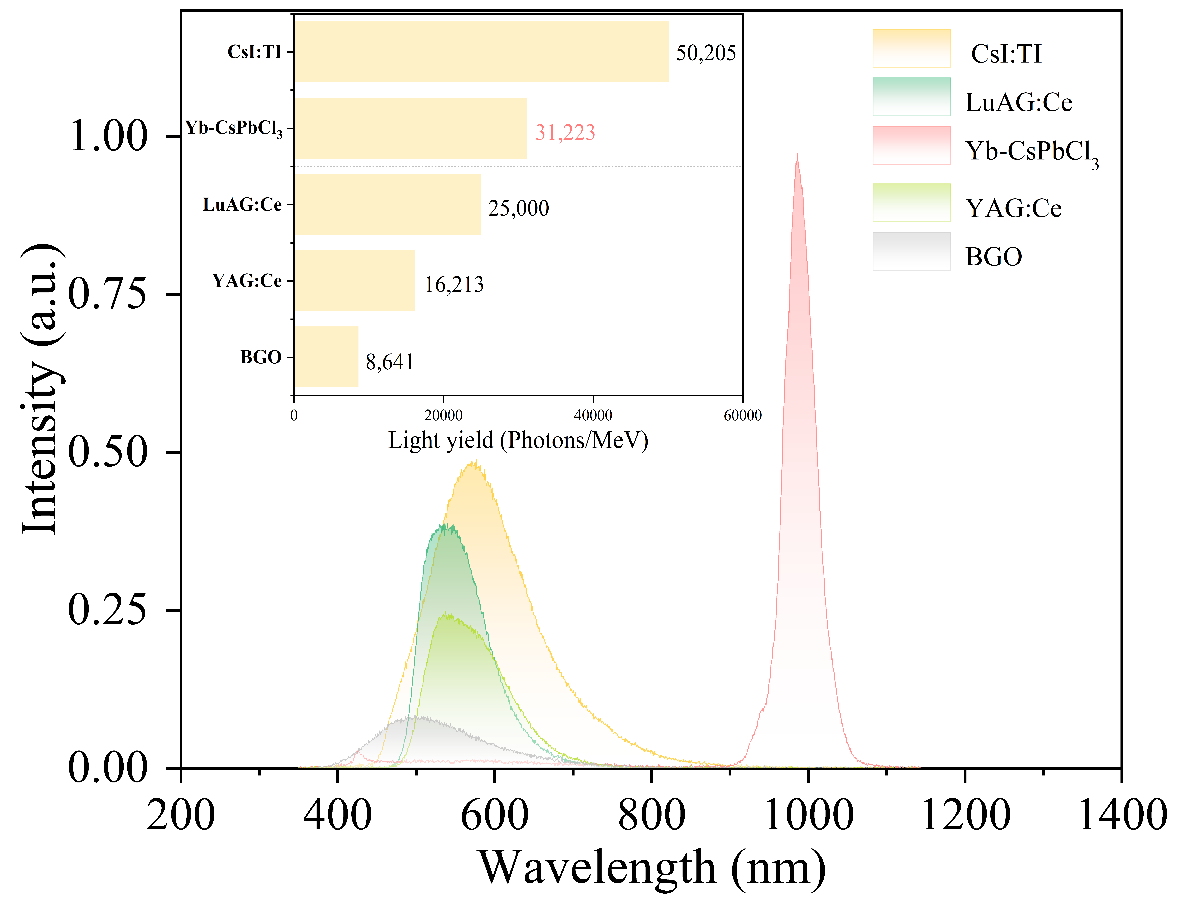


**Figure S11** The RL spectra of various scintillators with the same thickness, measured under identical conditions. The inset shows the relative light yield of these scintillators, calculated by comparing them to the reference sample.


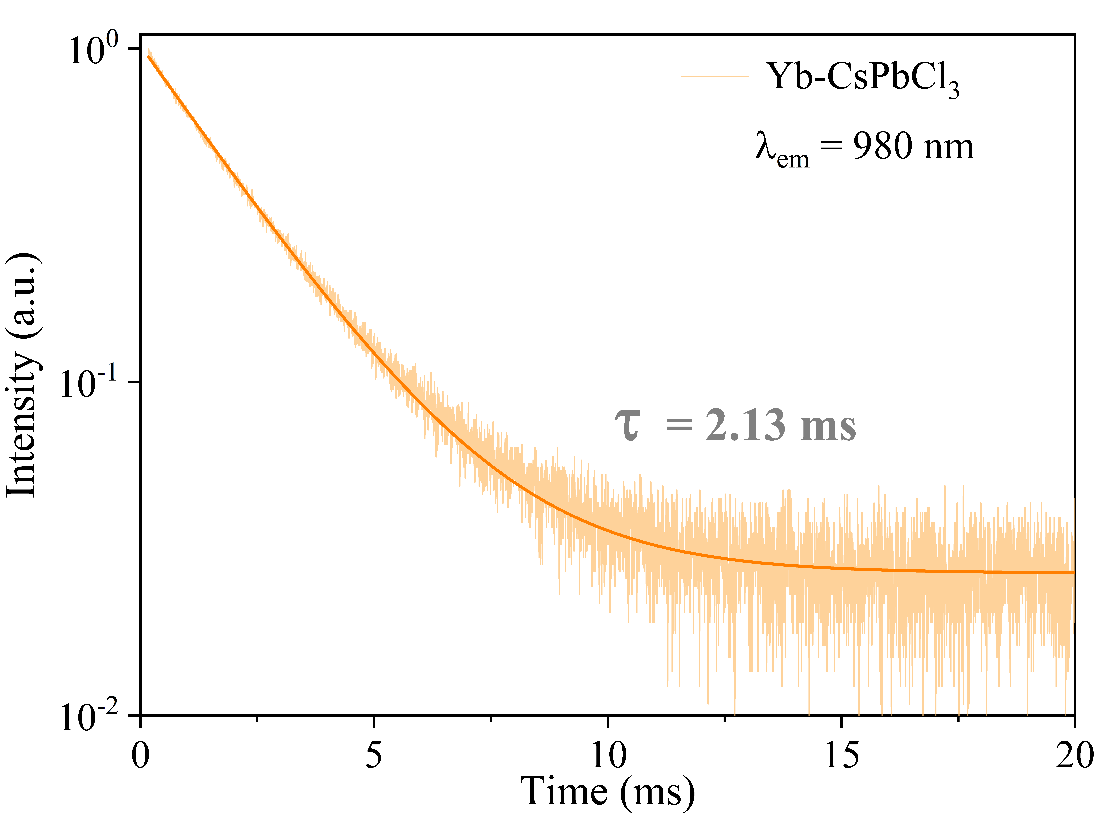


**Figure S12** TRPL spectrum of Yb-CsPbCl_3_ at room temperature (λ_em_ = 980 nm).


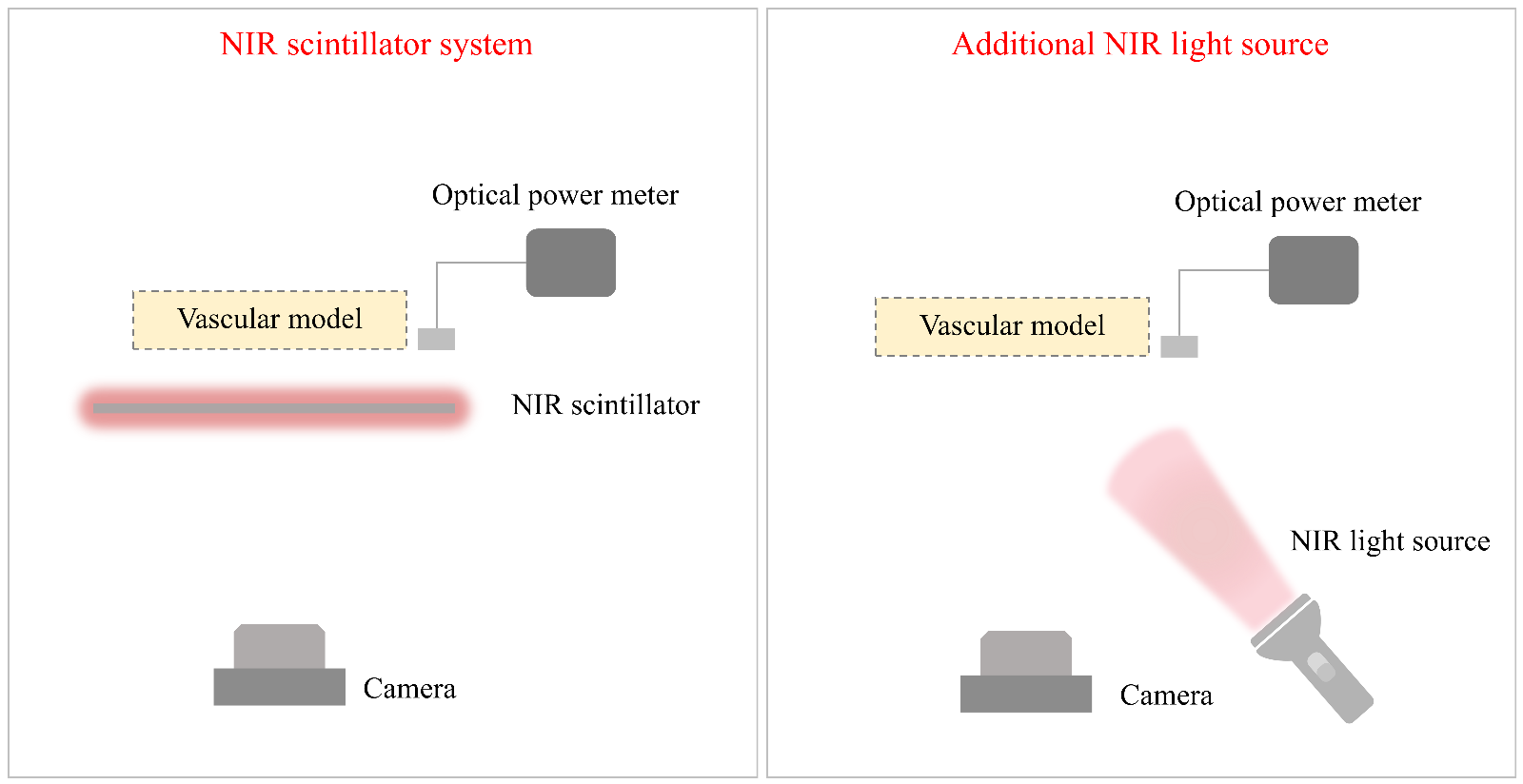


**Figure S13** The NIR scintillator system and an additional NIR light source were used for the NIR-only imaging experiment.


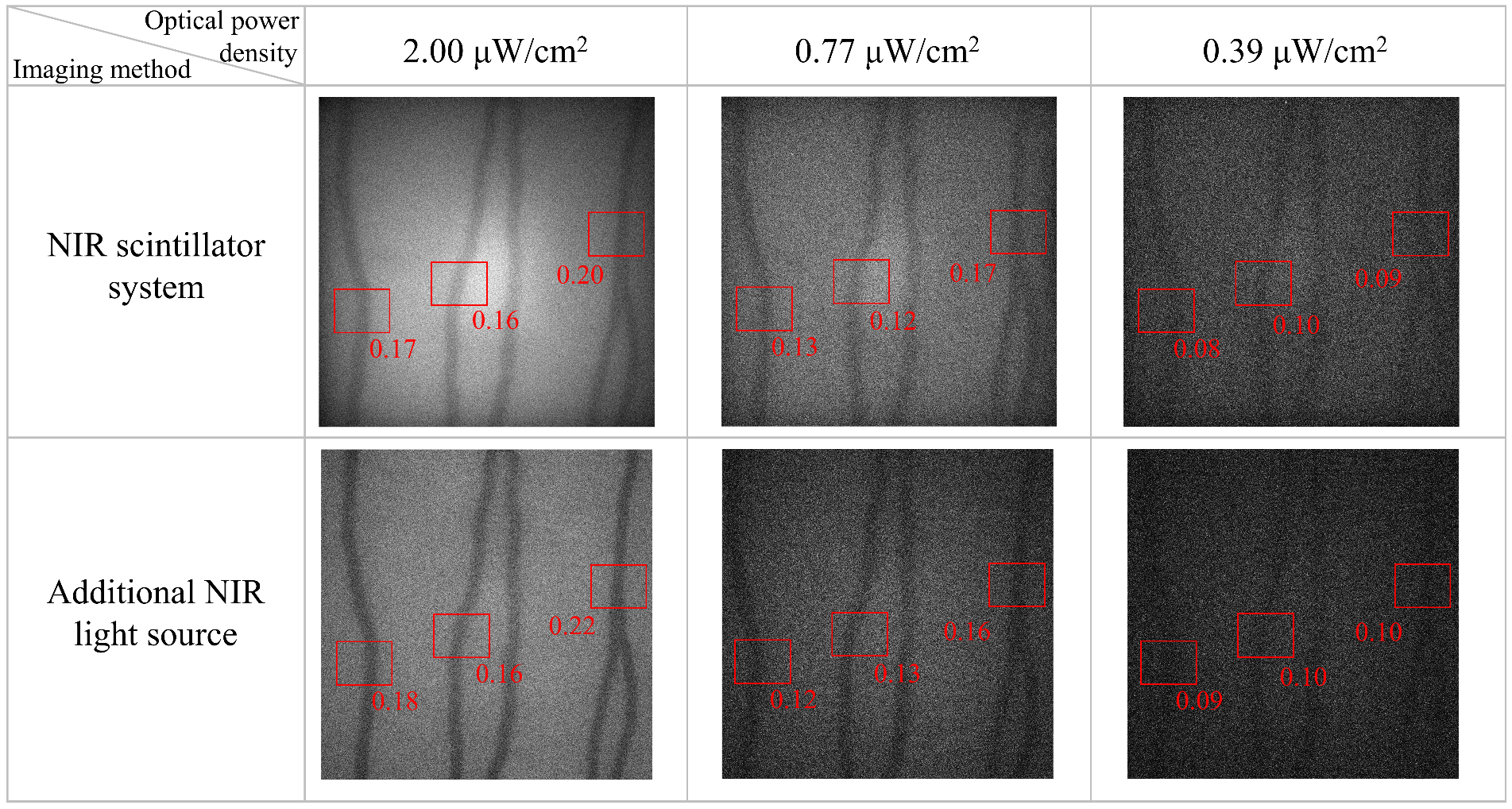


**Figure S14** Three sets of NIR-only images of the vascular model were obtained under different power densities using two imaging methods. The red numbers in the figure indicate the image contrast for the corresponding ROI.


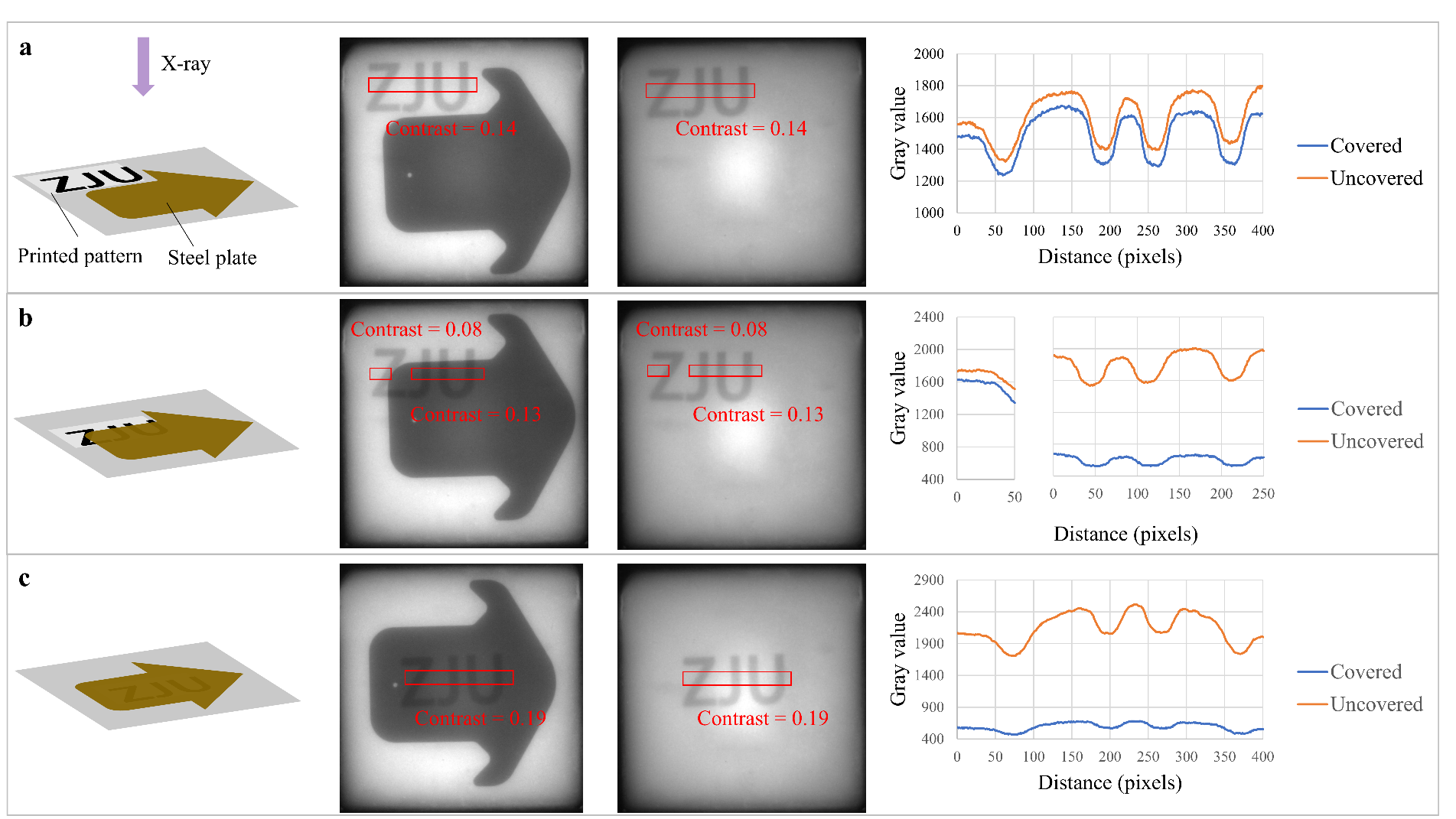


**Figure S15** The comparison of NIR imaging quality between non-uniform and uniform emission of the NIR scintillator. Panels a, b, and c compare NIR imaging under three different occlusion conditions to the no-occlusion scenario. The red numbers in the figure indicate the image contrast for the corresponding ROI. The graph on the right displays the gray value curve for these ROI.


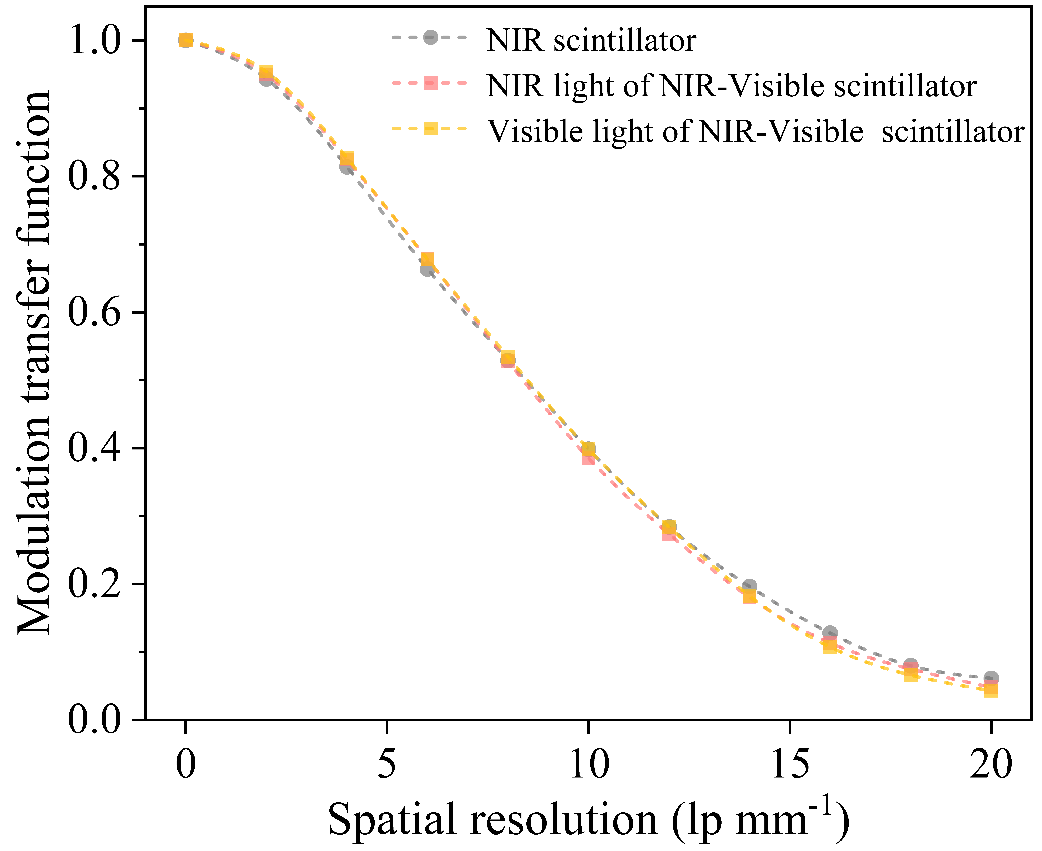


**Figure S16** The comparison of imaging spatial resolution between the NIR scintillator and the NIR-visible double-emission scintillator of equal thickness. The three MTF curves represent the NIR scintillator, the NIR light from the double-emission scintillator, and the visible light from the double-emission scintillator.


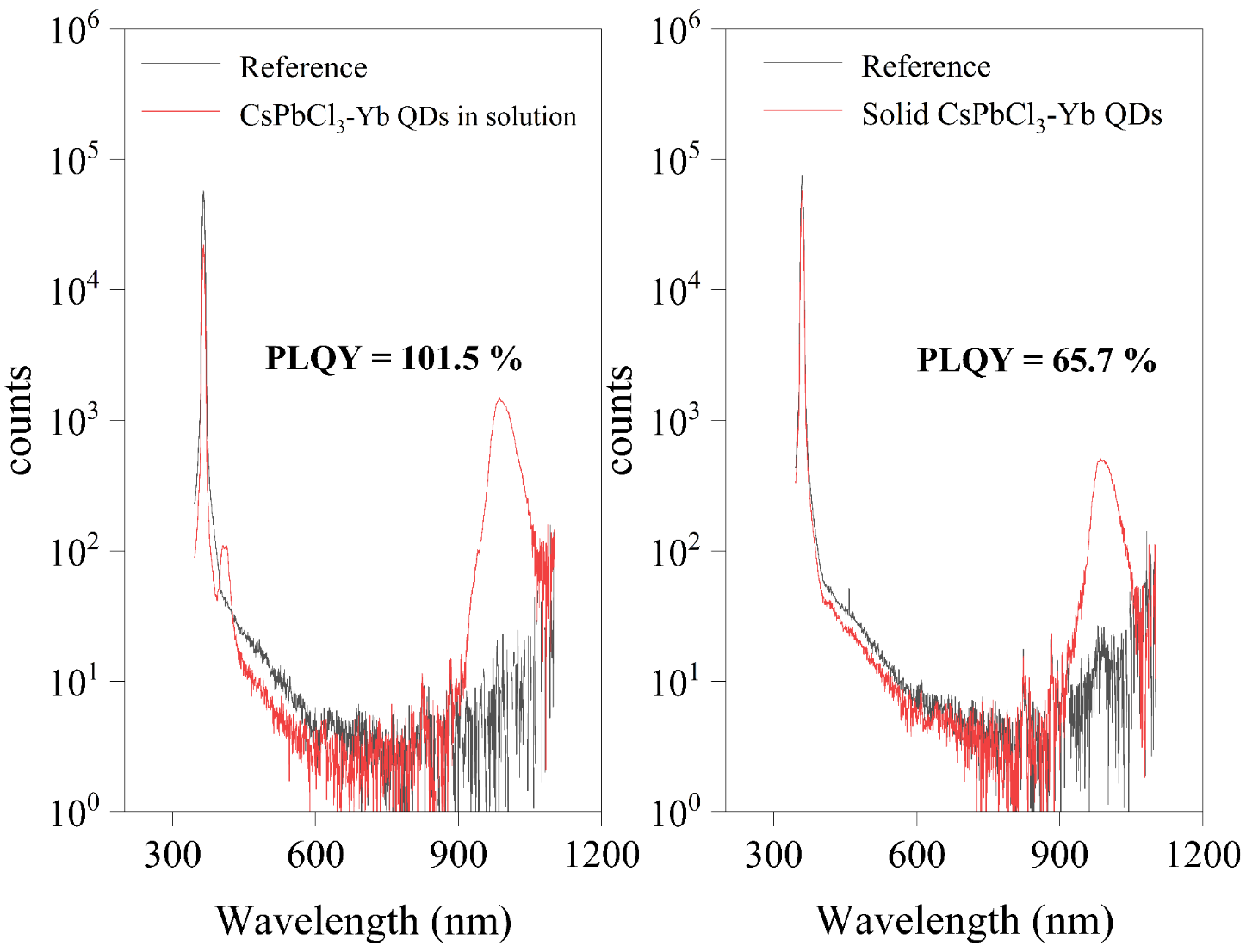


**Figure S17** The same Yb-CsPbCl_3_ QDs material was dispersed in toluene and formed into solid samples, allowing for the comparison of the measured PLQY data.


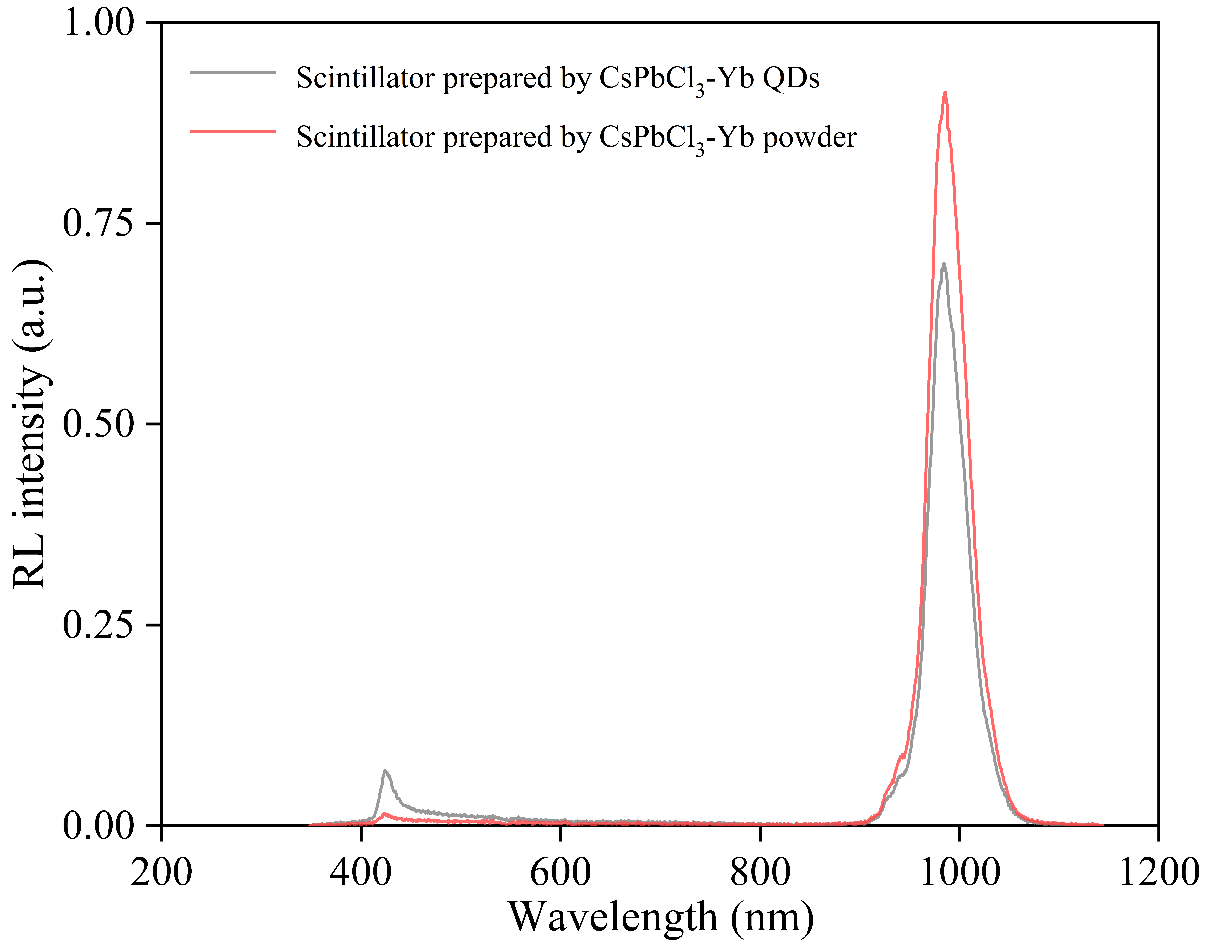


**Figure S18** The RL intensity of scintillators of equal thickness made from Yb-CsPbCl_3_ QDs and Yb-CsPbCl_3_ microcrystalline powder was compared.
